# The Effects of Postoperative Astaxanthin Administration on Nasal Mucosa Wound Healing

**DOI:** 10.3390/jcm8111941

**Published:** 2019-11-11

**Authors:** Lavinia-Gianina Manciula, Cristian Berce, Flaviu Tabaran, Veronica Trombitaș, Silviu Albu

**Affiliations:** 1 2nd Department of Otolaryngology, Iuliu Hatieganu University of Medicine and Pharmacy, 8 Victor Babes Street, 400012 Cluj-Napoca, Romania; veronicatrombitas@gmail.com (V.T.); silviualbu63@gmail.com (S.A.); 2 Department of Experimental Medicine, Iuliu Hatieganu University of Medicine and Pharmacy, 8 Victor Babes Street, 400012 Cluj-Napoca, Romania; cristian.berce@umfcluj.ro; 3 Pathology Department, University of Agricultural Sciences and Veterinary Medicine, 400372 Cluj-Napoca, Romania; flaviu_tabaran@yahoo.com

**Keywords:** nasal mucosa healing, astaxanthin, dexamethasone, synechia, epithelial thickness, subepithelial thickness, subepithelial fibrosis

## Abstract

Background: Wound healing of the nasal mucosa after endoscopic sinus surgery (ESS) is frequently complicated by scaring and consequently recurrences are encountered. Methods of optimizing results have been sought. In the present study we evaluated the effects of a powerful antioxidant, astaxanthin, on nasal mucosa healing after surgery, comparing it to the extensively studied properties of dexamethasone. Materials and Methods: 63 Wistar rats were used. The nasal mucosa from one side was damaged employing the brushing method. They were randomly divided into three experimental groups, one treated with astaxanthin, the second treated with dexamethasone and the third one acted as the control and was given normal saline. The rats were killed on days 5, 14 and 28 following injury. We observed the temporal evolution of the wound healing process and quantified the results by assessing four parameters: the epithelial thickness index (ETI), the subepithelial thickness index (STI), the goblet cell count and the subepithelial fibrosis index (SFI). Results: At 28 days, the ETI was significantly lower in the astaxanthin group (*p* < 0.05) compared to the other two groups. The STI was also lower in the astaxanthin group (*p* < 0.05), but comparable to the dexamethasone group at 28 days. The goblet cell count was higher in the astaxanthin group. The SFI had similar results in both dexamethasone and astaxanthin groups, with lower values compared to the control group. In the astaxanthin group there was no synechia formation. Conclusion: Astaxanthin given in the post injury period significantly decreases fibrosis, inhibits synechia development and significantly decreases subepithelial fibrosis. Moreover, it has no general or local toxic effects.

## 1. Introduction

Endoscopic sinus surgery (ESS) is considered the optimal management strategy for chronic rhinosinusitis resistant to medical treatment. ESS outcomes are reported to be extremely encouraging [1,2]. Nevertheless, recurrent disease requiring revision surgery is still being reported in 8% to 38% of patients [3,4]. Common findings in revision surgery are adhesions and sinus ostium stenosis [3,4]. Wound healing of the nasal mucosa succeeding ESS is a multifaceted process, often complicated by excessive scar formation and adhesions [4,5,6]. In this respect, methods of optimizing surgical results have been sought [7]. 

Since experimental studies are strictly restricted in humans, animal models became indispensable for apprehending the wound healing process [8]. The natural wound healing involves several stages, starting with inflammation, granulation tissue formation, reepithelization and remodeling of the newly formed epithelial tissue [5,6,8]. Rabbits or sheep have been previously defined as experimental models of nasal wound healing [9,10,11]. However, lately the rat model has been announced due to numerous benefits, including histology of the respiratory epithelium comparable to humans, small dimensions and faster wound healing compared to humans, rendering the study of the wound healing process much easier [8,12].

Postoperative wound healing is a complex process and encompasses three stages: inflammation, granulation formation and tissue remodeling [5,6]. The whole process is modulated by several factors, including reactive oxygen species ROS [13,14]. Reactive oxygen species (ROS) are byproducts of normal cellular metabolism that, among other functions, are also involved in all stages of wound closure [13,14,15,16]. During the first phase of inflammation, ROS play a key role in host defense against invading bacteria through local cell signaling, attracting neutrophils and macrophages to the injured site [13,14,15,16]. Moreover, ROS promote angiogenesis by stimulating endothelial cell division and migration, thus promoting blood vessel formation [14,15,16]. Angiogenesis is the key factor in granulation tissue formation. Finally, ROS trigger pro-matrix metalloproteinase-2 and induce cell motility, key factors in tissue remodeling [15]. However, overexposure to ROS, called oxidative stress, has an unfavorable effect on wound closure [13,14,15]. Hence, a precise balance between ROS and antioxidative mediators is essential for complete wound healing.

Astaxanthin is a xanthophyll carotenoid found naturally in various living organisms, mainly in the oceanic surroundings [17,18,19,20]. Astaxanthin is a powerful quencher of singlet oxygen [17]. Thus, it is able to regulate oxidative stress, acting as a potent antioxidant [17,18,19,20]. Astaxanthin cannot be manufactured by humans, and consequently must be picked up through their diet [17,21]. It has a reddish color and is usually found in microalgae, seafood, fish, quails and flamingos. Its main isomer (3S, 3S’) is produced by *Hematococcus pluvialis*, a green alga known for its high content in astaxanthin [20,21]. Astaxanthin bears a polar–nonpolar–polar configuration, which permits a perfect fit into the cellular membrane width [17,18,19,20,21]. This affords the cellular membrane’s lipid barrier a greater protection against peroxidation. Many experimental studies on various animals have demonstrated the efficacy of astaxanthin in cardiovascular diseases, diabetes mellitus, hypertension, and the ischemia-reperfusion myocardial model [17,18,19,20,21].

A study conducted by Mizuta et al. [22], demonstrated that astaxanthin had a positive effect by decreasing inflammation during the initial phase of the vocal fold wound healing, and increasing the expression of procollagen type I and bFGF on postoperative day I. The astaxanthin-treated group showed a significant increase in hyaluronic acid deposition and attenuation of the contraction of the lamina propria [22]. These findings led to the conclusion that astaxanthin has a positive effect on preventing scaring after laryngeal interventions.

A recent experimental study on the rat model concluded that systemic administration of dexamethasone following mucosal damage might decrease subepithelial edema, goblet cell hyperplasia and adhesion development [12]. We aimed in our study to assess the effects of astaxanthin on the nasal mucosa healing process succeeding surgery, comparing its effects to those of dexamethasone.

## 2. Materials and Methods

### 2.1. Animal Preparation

The present study was approved by The Iuliu Hatieganu University of Medicine and Pharmacy Ethics Committee and the experiments were performed according to the Guidelines of the National Institutes of Health and the Declaration of Helsinki. The animals were kept in a 21 °C temperature environment, were supplied with food and water ad libitum, and had a day–night cycle of 12/12 h.

In the present study, 63 Wistar rats were included, both male and female, weighing about 300 g each. Anesthesia was achieved by intraperitoneal (IP) injection of xylazine hydrochloride (4.5 mg/kg) and ketamine hydrochloride (45 mg/kg). The nasal mucosa from one side was damaged employing the brushing method. An interdental brush (10 mm) inserted through the nostril induced a mechanical wound [8]. Afterwards, the rats were randomly divided into 3 experimental groups, the first one treated with astaxanthin by oral gavage (100 mg/kg daily for 3 days). Dexamethasone was administered IP to the second group (0.15 mg/kg daily for 7 days). The third group acted as the control and was given normal saline (0.15 mg/kg daily for 7 days). Seven rats from each group were killed in the postoperative days 5, 14 and 28.

For the histological examination, the head was immediately harvested and fixed for 72 h in 10% neutral-buffered formalin. After the complete fixation, the heads were cleaned of connective tissue and skin and decalcified for three days in a 1:1 mixture of formic and hydrochloric acid (8%) [23]. When decalcification was completed, the tissues were transversely trimmed in four planes following the previously described technique by Kittel et al. [24], at the following localizations: (1) Posterior part of upper incisors, (2) incisive papilla, (3) second palatine crest, and (4) first molar teeth. After this step, the samples were subsequently immersed overnight on a stirrer in the same mixture of formic and hydrochloric acid as described above [23,24]. Afterwards, the samples were dehydrated using an ethylic alcohol gradient (70%, 80%, 90%, 95% and 100%), clarified in xylene and embedded with the rostral faces down in paraffin wax. Tissue sections were cut from each paraffin block at 4 µm thickness and routinely stained with hematoxylin and eosin (H&E), Masson’s trichrome and Toluidine blue [23]. Finally, the histological slides were examined using an Olympus BX41 microscope. Bright field images and all morphometric evaluations were taken with an Olympus UC30 digital camera and processed using the Stream basic program. The pathologist who performed the histological examination was blinded to the study groups.

### 2.2. Histological Analysis

In order to evaluate the outcome of the different treatments on the wound healing process, at the septal wound site various features were assessed, such as inflammatory cell infiltration, goblet and ciliary cell development, epithelial thickness, subepithelial thickness, subepithelial fibrosis and the incidence of adhesions [8,12]. As previously described, different histological indices were employed in our analysis [8,12]:

The epithelial thickness index (ETI) value was obtained by calculating the ratio of the averaged height of the newly regenerated epithelium to the height of the averaged epithelium from the contralateral side, measured by taking the average distance between the basement membrane and apical surface of the epithelium from three mucosal sampling areas in H&E-stained sections at 100× magnification. An ETI value > 1.0 is considered to represent hypertrophic epithelium [8,12]. 

The subepithelial thickness index (STI) value was obtained by calculating the ratio of the averaged height of the newly regenerated subepithelium to the height of the averaged subepithelium from the contralateral side, measured by taking the average length of lamina propria from three mucosal sampling areas in H&E-stained sections at 100x magnification. An STI value > 1.0 is considered to represent hypertrophic subepithelium [8,12]. 

The degree of fibrosis was expressed as the subepithelial fibrosis index (SFI), evaluated on relative intensities of Masson’s trichrome-stained sections at 20× magnification. The collagen staining intensity was measured from three subepithelial areas on the injured and contralateral sides of the nasal septum. An SFI > 1.0 represents increased collagen fibrosis [8,12]. 

The number of goblet cells was counted in the Masson’s trichrome-stained sections at 100× magnification at the level of the wound area.

### 2.3. Statistical Analysis

Due to the fact that samples were small and the variables were not normally distributed, comparisons between the groups were conducted with the Kruskall–Wallis non-parametric test. Median and interquartile ranges were used in the analysis and the results were represented graphically. Measurements were also made in time. Therefore, the MANOVA test with repeated measurements was employed. The statistical analysis was performed using SPSS software (SPSS Inc.ver.20.0, Chicago, IL, USA). A *p* value < 0.05 was considered significant.

## 3. Results

### 3.1. Histologic Modifications in Relationship to Time

We followed the temporal healing pattern of the nasal mucosa in the three different experimental groups, describing the histological aspects, with focus on inflammation, epithelial regeneration and fibrosis evolution. A recent paper demonstrated that important amounts of ROS are formed in the course of the early stage of wound healing, namely in the first three days succeeding injury [25]. Thus, this period might be critical in controlling ROS levels and this is why we administered astaxanthin only during the first three days of the experiment [22,25].

**Figure 1:**
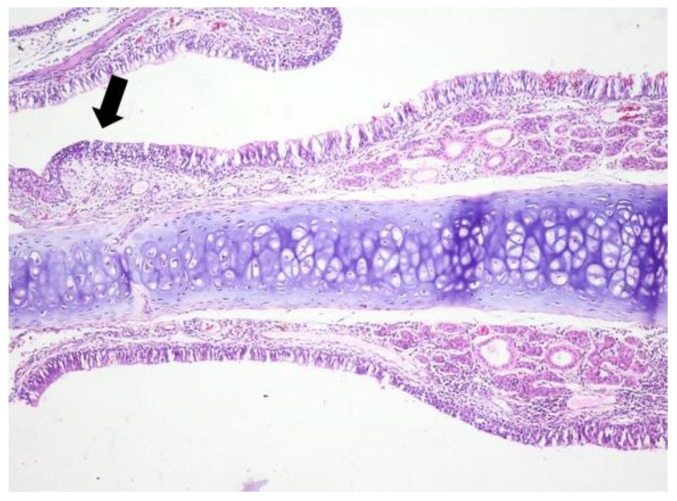
Light micrographs of the rat’s nasal septum, control group, at 5 days after the intranasal intervention. Bilateral, minimal, diffuse infiltration of the lamina propria of the mucosa covering the nasal septum by few mononuclear cells. Focally, the lamina propria is expanded by fibrous connective tissue and the covering mucosa is infiltrated by lymphocytes, macrophages and plasma cells (arrow); H&E, ob ×10.

**Figure 2:**
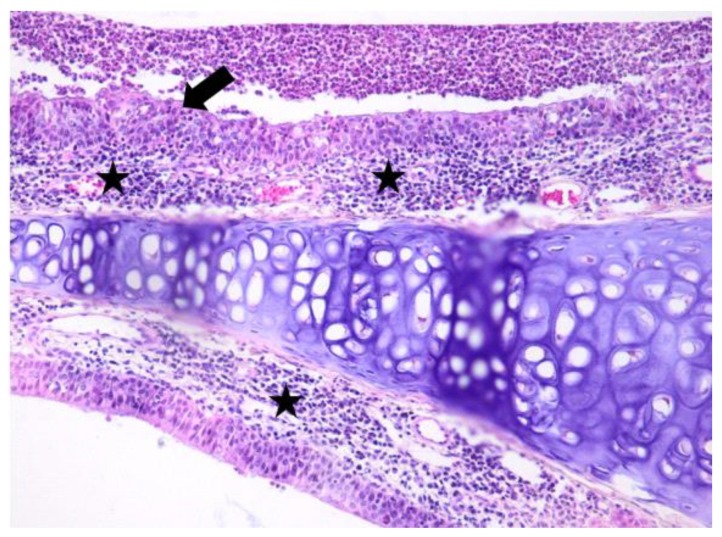
Light micrographs of the rat’s nasal septum, dexamethasone group, at 5 days after the intranasal intervention. The septal nasal mucosa is covered by an abundant purulent material, is focally hyperplastic, with occasional squamous metaplasia (arrow). The lamina propria is expanded and the nasal glands are replaced by lymphocytes, macrophages and plasma cells (black star). H&E, ob ×20.

**Figure 3:**
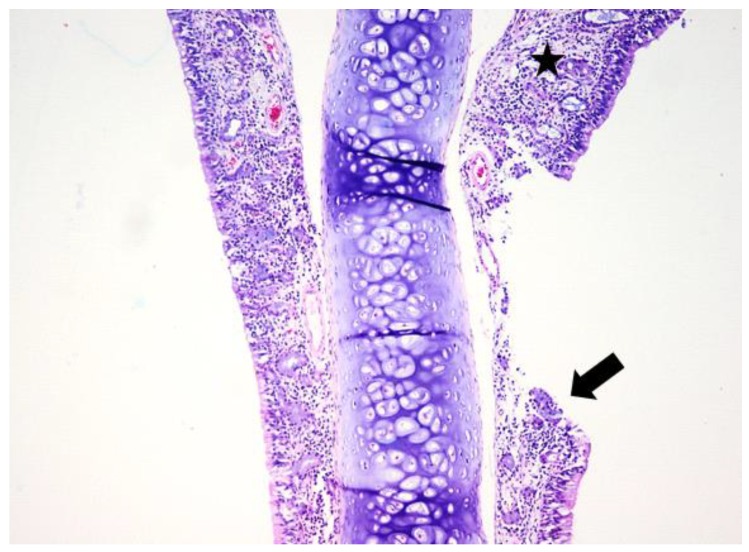
Light micrographs of the rat’s nasal septum, astaxanthin group, at 5 days after the intranasal intervention. The nasal mucosa is disrupted with few cell debris admixed with mucus adherent on the edges of the defect (arrow). The lamina propria of the margins is expanded by mononuclear cells admixed with neutrophils and mild edema (arrow); H&E, ob ×10.

On the fifth day, the histological pattern in the control group displayed areas of minimal hyperplasia, moderate squamous metaplasia and goblet cell growth with a focal pseudoglandular pattern; diffuse inflammatory cell infiltrate composed of lymphocytes, macrophages, plasma cells, neutrophils and eosinophils (Figure 1). In the dexamethasone group, diffuse suppurative exudate, minimal squamous metaplasia and scarce leukocyte infiltrate were noticed (Figure 2). The astaxanthin-treated group expressed seromucous gland hyperplasia, discreet hyperplasia of the respiratory epithelium, squamous metaplasia and minimal fibrosis (Figure 3).

**Figure 4:**
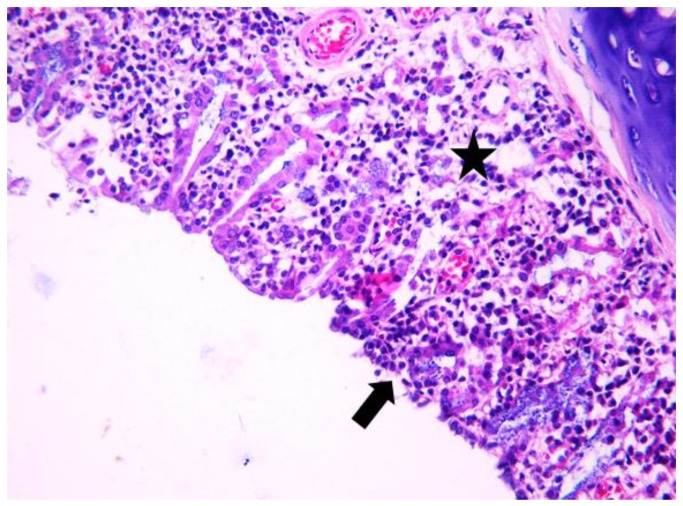
Light micrographs of the rat’s nasal septum, control group, at 14 days after the intranasal intervention. Diffuse infiltration of the lamina propria of the mucosa covering the nasal septum by many lymphocytes admixed with macrophages and few plasma cells (black star). The covering mucosa is occasionally erosive (arrow); H&E, ob ×40.

**Figure 5:**
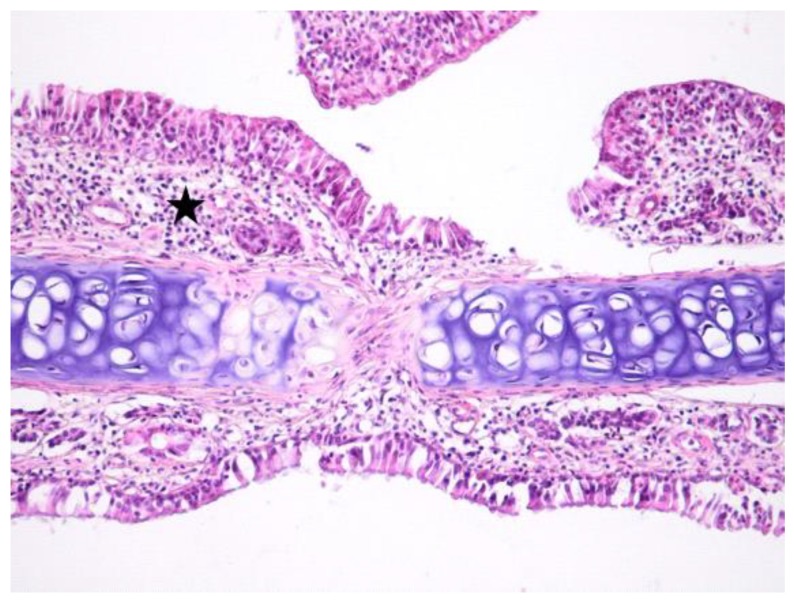
Light micrographs of the rat’s nasal septum, dexamethasone group, at 14 days after the intranasal intervention. The lamina propria of the mucosa covering the nasal septum is moderately expanded and the nasal glands are replaced by lymphocytes, macrophages and plasma cells (black star). H&E, ob ×20.

**Figure 6:**
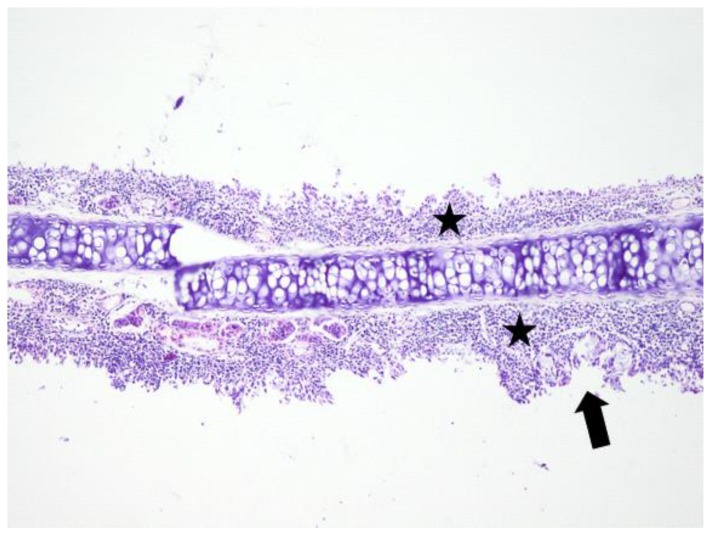
Light micrographs of the rat’s nasal septum, astaxanthin group, at 14 days after the intranasal intervention. Diffusely, the lamina propria of the mucosa covering the nasal septum is markedly expanded by many macrophages admixed with lymphocytes and few plasma cells (black star). The covering mucosa is focally erosive and occasionally hyperplastic (arrow); H&E, ob ×10.

On the 14th day, the histological pattern displayed squamous metaplasia with severe seromucous gland hyperplasia in the control group, and at the level of the epithelium, mononuclear cells infiltrate into the lamina propria (Figure 4). In the dexamethasone group, there is complete regeneration of the respiratory mucosa, with mixed perilesional inflammation and subtle metaplasia, suppurative exudate. Subepithelial fibrosis and inflammation are minimal (Figure 5). In the astaxanthin group, there is complete regeneration of the respiratory mucosa, with discreet mixed inflammation, together with areas of mucous hyperplasia, squamous metaplasia and moderate suppurative exudate. There is also severe perilesional seromucous gland hyperplasia and subepithelial fibrosis (Figure 6).

**Figure 7:**
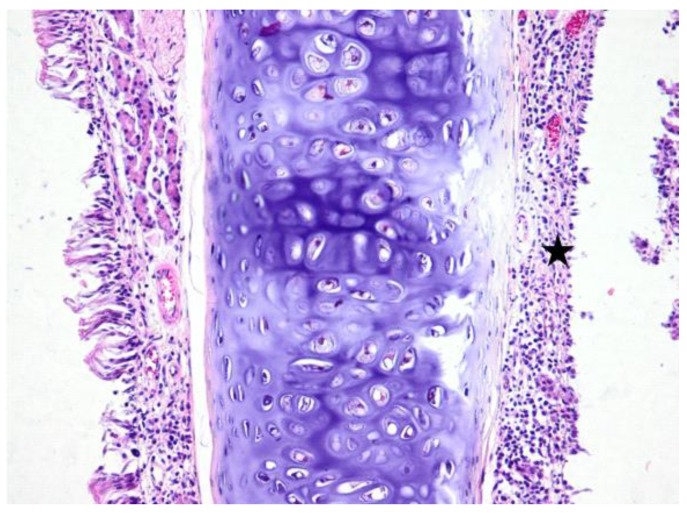
Light micrographs of the rat’s nasal septum, dexamethasone group, at 28 days after the intranasal intervention. Unilaterally the lamina propria of the mucosa covering the nasal septum is moderately expanded and the nasal glands are replaced by lymphocytes, macrophages and plasma cells (black star). The covering mucosa is focally erosive; H&E, ob ×20.

**Figure 8:**
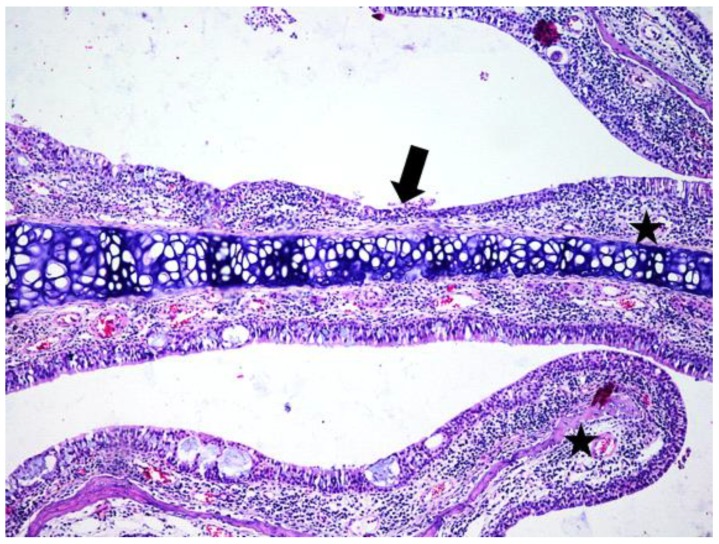
Light micrographs of the rat’s nasal septum, astaxanthin group, at 28 days after the intranasal intervention. Diffusely the lamina propria of the mucosa covering both the nasal septum and nasal conchae is expanded by many lymphocytes admixed with macrophages, few plasma cells (black star) and edema. The covering mucosa is focally excavated (arrow) and covered by squamoid epithelium; H&E, ob ×10.

On day 28 in the control group, the epithelium shows areas of mucous hyperplasia, perilesional seromucous glands and goblet cell hyperplasia. There is moderate subepithelial fibrosis and inflammation associated with the areas of mucous hyperplasia. In the dexamethasone group, a focal absence of the respiratory mucosa and minimal subepithelial fibrosis were noticed. There is severe perilesional glandular atrophy and seromucous gland regeneration (Figure 7). In the astaxanthin group, the examination revealed complete regeneration of the respiratory mucosa, with absent perilesional hyperplasia or metaplasia, with glandular regeneration and no subepithelial fibrosis (Figure 8). 

### 3.2. Index Changes in Relationship to Time

The descriptive statistic of the variables used is summed up in Table 1.

**Figure 9:**
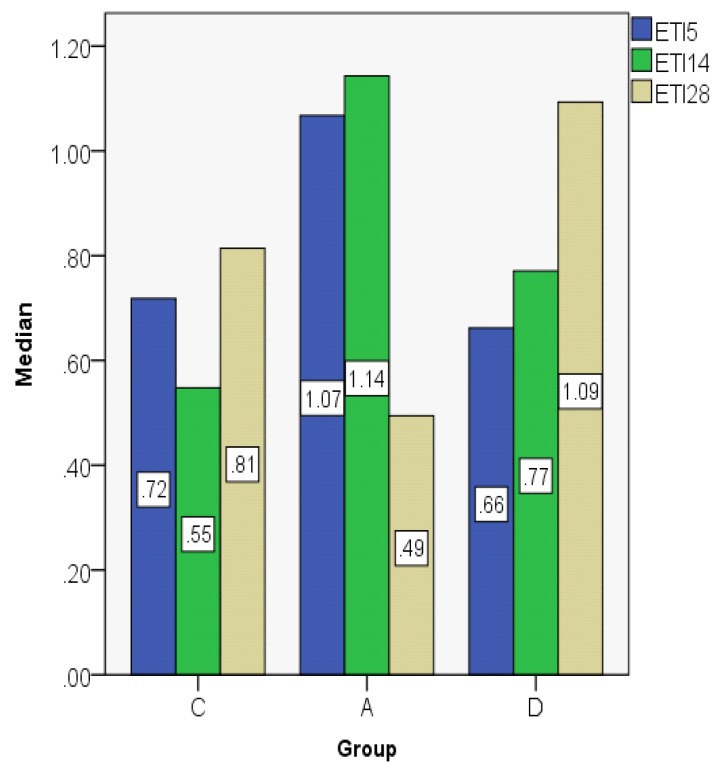
Epithelial thickness index (ETI) value distribution (median).

**Figure 10:**
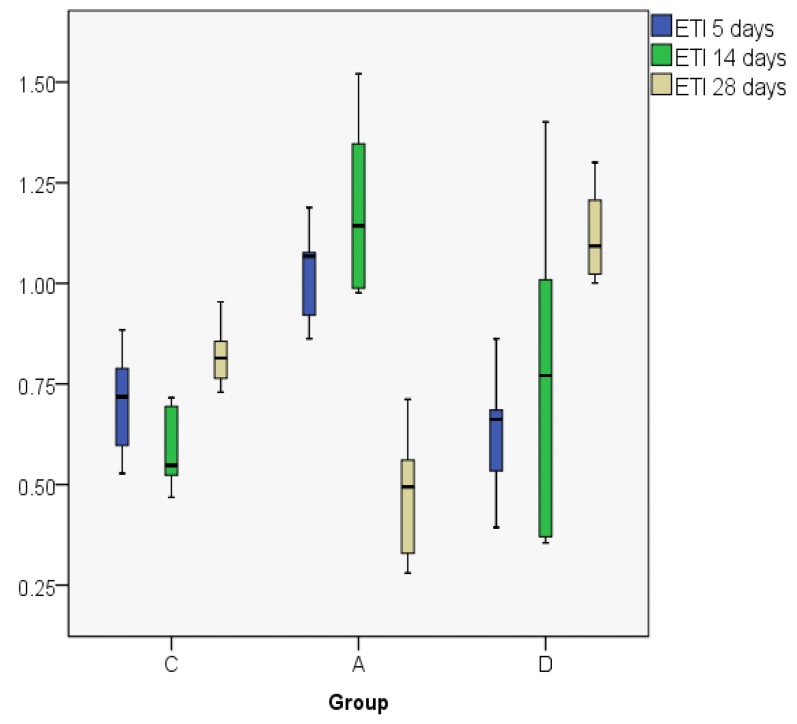
Epithelial thickness index (ETI) value distribution (boxplot representation).

ETI indices are presented in Figure 9; Figure 10: In the control group, the ETI value was higher on day 28 in comparison to day 5; in the dexamethasone group it displays an ascending trend, its value being higher on day 28. However, in the Astaxanthin group, ETI at day 28 had the lowest value when compared to the other groups, but also in comparison to day 5. In order to analyze the statistical differences between groups, we used the Kruskal–Wallis non-parametric test. When contemplating the results presented in Table 2, the differences are statistically significant.

**Figure 11:**
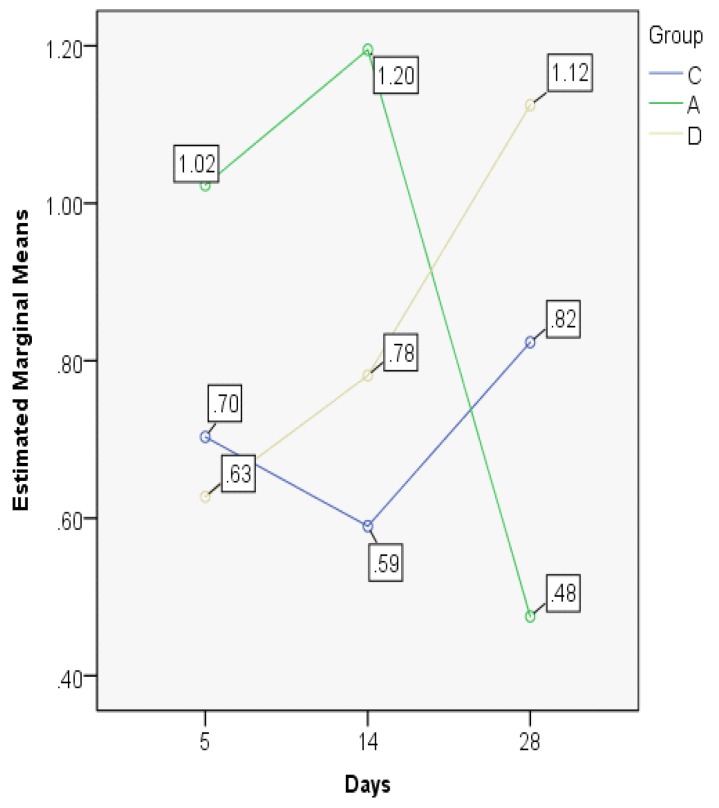
Epithelial thickness index (ETI) values, MANOVA test.

The significant statistical difference is also outlined by the MANOVA test, displaying important differences between the developments in time of the indices in the three experimental groups (Figure 11). The control and dexamethasone groups demonstrate a similar pattern of ETI evolution, opposed to the astaxanthin group where a significant drop on the 28th day is recorded (the lowest documented value).

**Figure 12:**
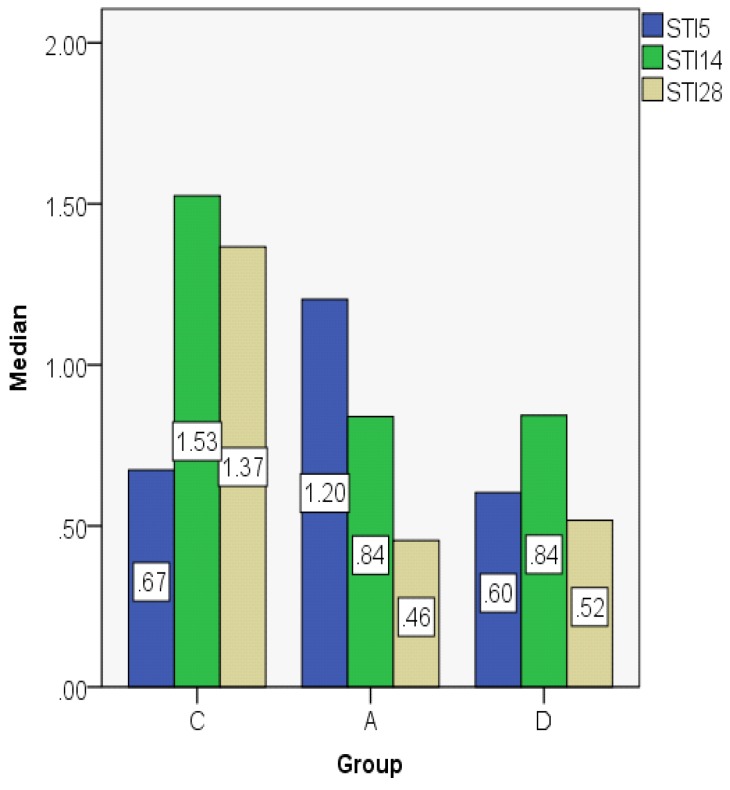
Subepithelial thickness index (STI) value distribution (median).

**Figure 13:**
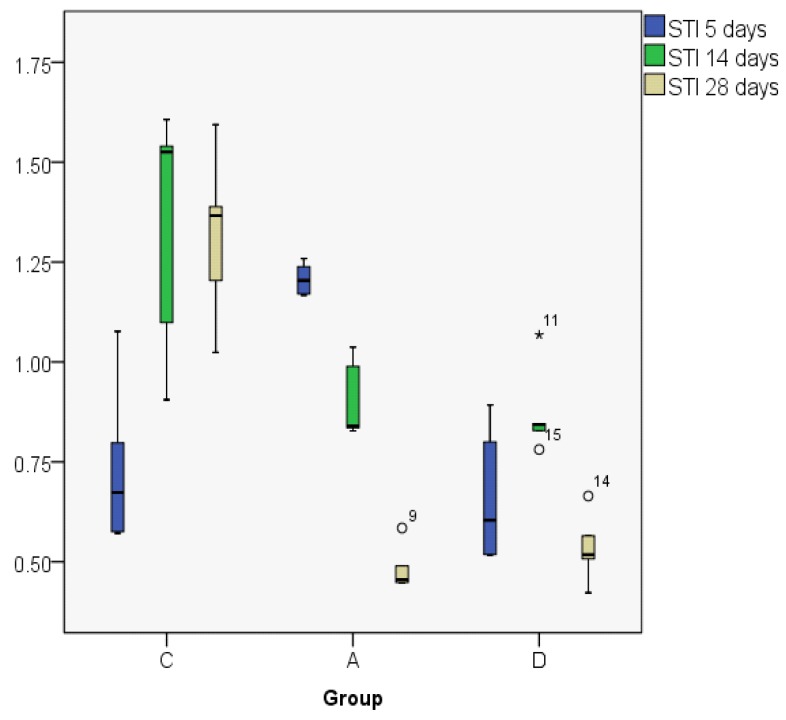
Subepithelial thickness index (STI) value distribution (boxplot representation).

The STI indices are comparable between days 14 and 28 in the control group, but in the astaxanthin and dexamethasone groups they decline on day 28, having comparable values (Figure 12; Figure 13).

**Figure 14:**
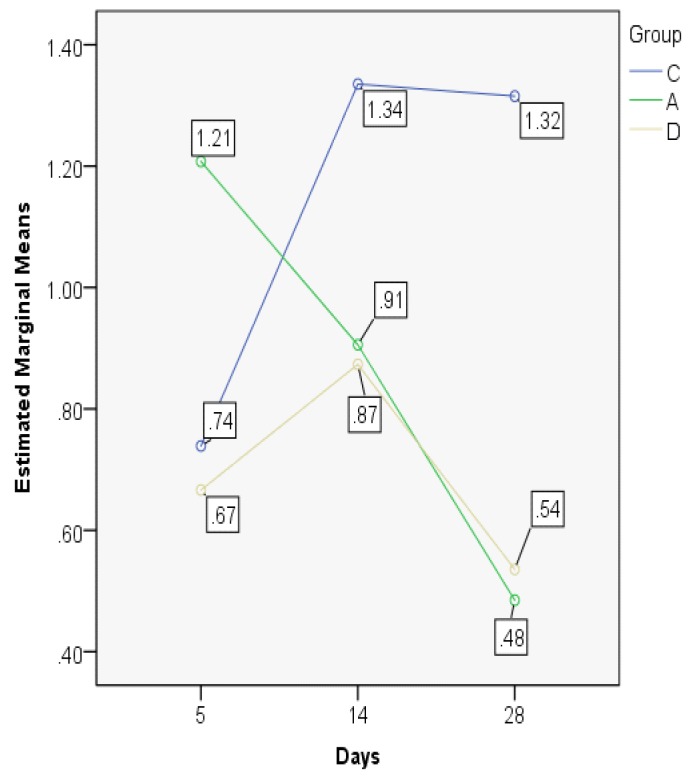
Subepithelial thickness index values, MANOVA test.

The MANOVA test showed significant differences between groups regarding the evolution in time, but also when comparing the three experimental groups. On day 5, there were similar results for the control and the dexamethasone groups, but significantly lower as compared to astaxanthin. The control group has an ascending trend, different from the dexamethasone and astaxanthin groups, both presenting a significant and constant decline on days 14 and 28 (Figure 14).

**Figure 15:**
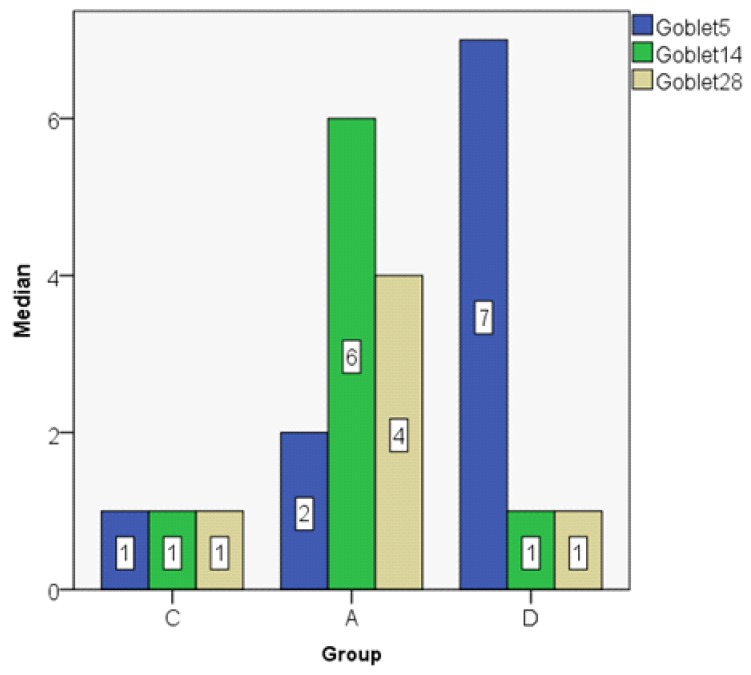
Goblet cell count (median).

**Figure 16:**
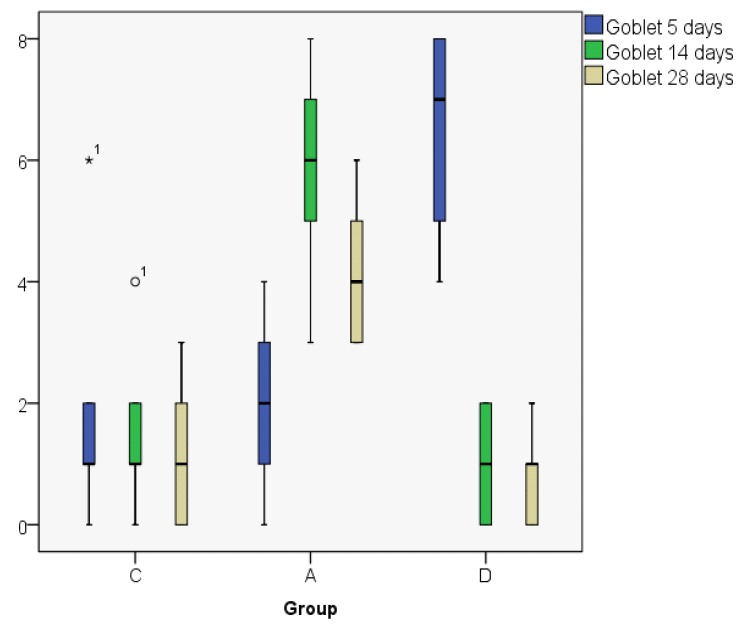
Goblet cell count (boxplot representation).

The goblet cell count shows a decreasing value on day 28 as compared to day 5 in the control and dexamethasone groups, but in the astaxanthin group the value was higher on day 28 when compared to day 5. On day 14, we obtained the same values both in the astaxanthin and the dexamethasone groups (Figure 15; Figure 16).

**Figure 17:**
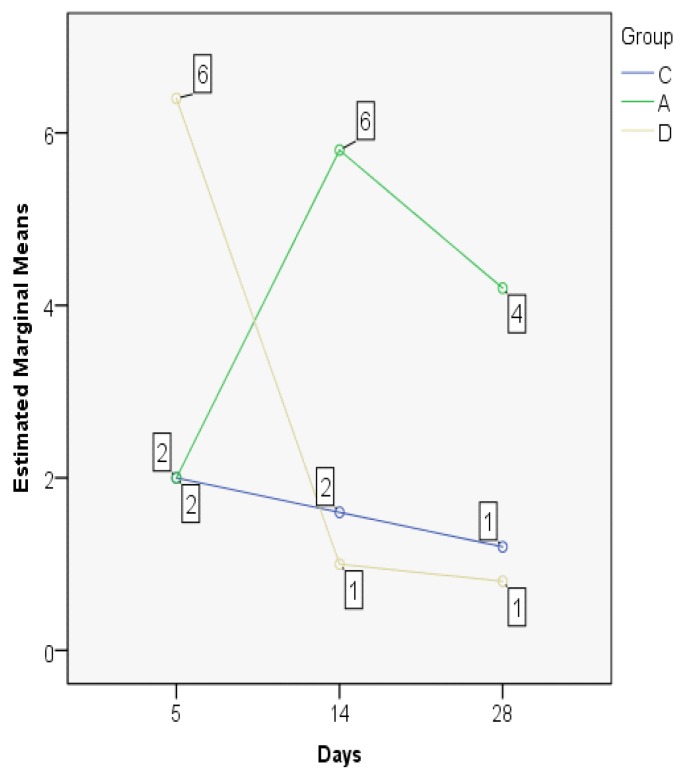
Goblet cell count, MANOVA Test.

The MANOVA test shows a significantly lower cell count on day 5 in the control group as compared to the dexamethasone group (*p* < 0.05). On day 14 there were no statistically significant differences between the control and dexamethasone groups, but there was a significant difference between the astaxanthin and the control groups, the astaxanthin group showing a higher goblet cell count than the control group (*p* < 0.05) (Figure 17).

**Figure 18:**
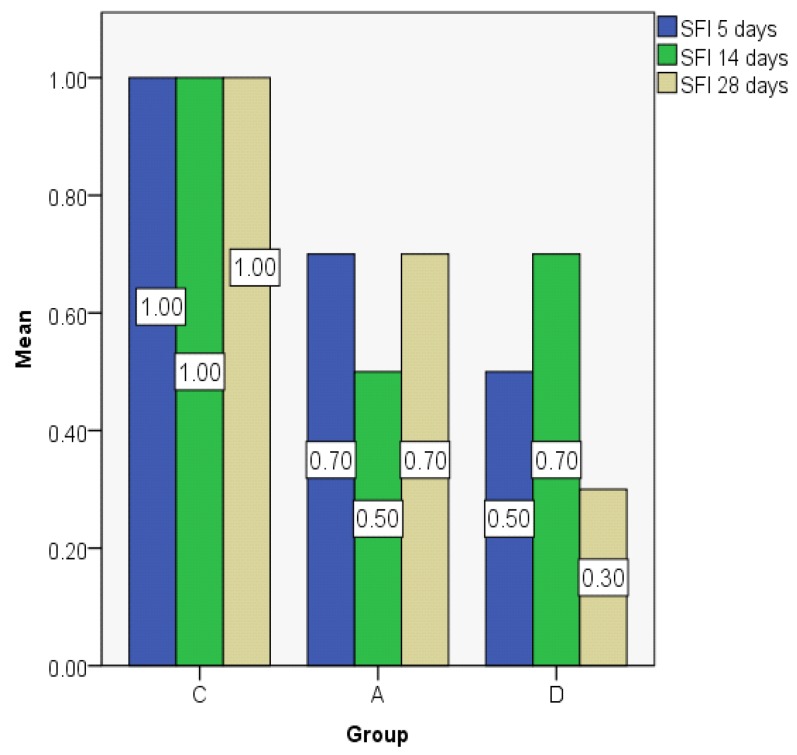
Subepithelial fibrosis index (SFI) value distribution expressed as the mean.

The subepithelial fibrosis index showed only small differences between groups, but for the dexamethasone group the value was significantly smaller than that of the control group on day 28 (Figure 18).

## 4. Discussion

The most common causes of surgical failure following ESS are synechia development and ostium stenosis [3,4]. Topical steroids have been long used to impede the development of recurrent nasal polyps [26]. Moreover, Jorissen et al. [27] have demonstrated that early postoperative topical steroids may even improve wound healing, especially in nasal polyposis. Dexamethasone impregnated stents were employed with considerable success in experimental models [28]. In a prospective, randomized study, Wright et al. [29] stated that preoperative and postoperative systemic corticoids improve considerably the objective and subjective outcomes. Moreover, in pediatric patients it is suggested that intraoperative dexamethasone decreases maxillary mucosa swelling and granulations [30]. However, it is acknowledged that systemic corticoid employment bears the risk of serious side effects, thus limiting its indications. In a recent experimental study on rats, it was demonstrated that systemic postoperative dexamethasone decreases edema, goblet cell hyperplasia and adhesions, but it seems to have a negative impact on mucociliary regeneration [12]. Lately, Testa et al. [31] confirmed that topical alpha-tocopherol acetate is competent to significantly improve the post-operative healing of elderly CRS patients deferred to ESS.

A previous study on wound healing of the vocal folds proved that ROS is mainly produced in the first 3 days following injury; therefore, this period is critical in oxidative stress adjustment [25]. Hence, in our experiment astaxanthin was provided in the first 3 days following injury of the septum.

In our study, in the astaxanthin-treated group there was moderate inflammation on day 5 at the level of the epithelium and lamina propria as compared to the dexamethasone group and the control group. On day 14, the respiratory mucosa was completely regenerated and the subepithelial fibrosis was moderate. On day 28, there was complete regeneration of the respiratory mucosa and the subepithelial fibrosis was absent. On microscopic examination of the turbinates and paranasal sinus area we could not observe any synechia or obvious atrophy. On the contrary, the control group displayed atrophy of the mucosa of the turbinates and epithelial atrophy and fibrosis in the paranasal sinuses. In the dexamethasone group the examination revealed turbinate atrophy and synechia formation. These findings indicate that the positive effect of astaxanthin leads not only to a decrease in fibrosis and synechia formation at the injury site, but it also has a positive influence on the mucosa of the surrounding areas. During the healing period, both the control and dexamethasone groups present an ascending trend of the epithelium thickness index. On the contrary, on the 28th day the astaxanthin group displays a marked reduction of the epithelium thickness. Our finding confirms a previous study, where astaxanthin significantly reduced vocal fold scarring. It also seems that the effect of astaxanthin is quite similar to that of dexamethasone regarding subepithelial fibrosis on day 28, making it a good alternative in the setting where corticosteroids are not indicated or tolerated. However, an interesting finding was the significant enhancement of goblet cell proliferation in the astaxanthin group. Since these cells display a normal pattern on histological examination, we can suppose that the mucociliary clearance is improved in these animals. 

However, there are also several limitations of our study: Definite conclusions cannot be drawn because of the restricted time span of our study, and the experiments were done on healthy, noninfected rats. We should expand our knowledge on wound healing in the context of induced inflammation and infection of the paranasal sinuses.

## 5. Conclusions

Despite its limitations, we have been able to demonstrate in an experimental setting that astaxanthin given in the post injury period significantly decreases fibrosis, inhibits synechia development and significantly decreases subepithelial fibrosis. Moreover, it has no general or local toxic effects.

## Figures and Tables

**Table 1:** Descriptive statistics of the variables used.

	Group	Mean	Median	Std. Deviation	Minim	Maxim	Range	IQR
Goblet5	C	2.00	1.00	2.345	0	6	6	4
	A	2.00	2.00	1.581	0	4	4	3
	D	6.40	7.00	1.817	4	8	4	4
Goblet14	C	1.60	1.00	1.517	0	4	4	3
	A	5.80	6.00	1.924	3	8	5	4
	D	1.00	1.00	1.000	0	2	2	2
Goblet28	C	1.20	1.00	1.304	0	3	3	3
	A	4.20	4.00	1.304	3	6	3	3
	D	0.80	1.00	0.837	0	2	2	2
ETI5	C	0.7032	0.7182	0.14353	0.53	0.88	0.36	0.27
	A	1.0231	1.0674	0.13079	0.86	1.19	0.33	0.24
	D	0.6274	0.6620	0.17541	0.39	0.86	0.47	0.31
ETI14	C	0.5898	0.5478	0.10927	0.47	0.72	0.25	0.21
	A	1.1950	1.1429	0.23580	0.98	1.52	0.54	0.45
	D	0.7812	0.7708	0.44348	0.35	1.40	1.05	0.84
ETI28	C	0.8236	0.8142	0.08743	0.73	0.95	0.22	0.16
	A	0.4753	0.4942	0.17553	0.28	0.71	0.43	0.33
	D	1.1247	1.0930	0.12677	1.00	1.30	0.30	0.24
STI5	C	0.7389	0.6732	0.21001	0.57	1.08	0.50	0.36
	A	1.2077	1.2038	0.04060	1.17	1.26	0.09	0.08
	D	0.6663	0.6038	0.17112	0.52	0.89	0.38	0.33
STI14	C	1.3352	1.5252	0.31325	0.91	1.61	0.70	0.57
	A	0.9056	0.8392	0.09938	0.83	1.04	0.21	0.18
	D	0.8732	0.8434	0.11184	0.78	1.07	0.29	0.15
STI28	C	1.3153	1.3664	0.21380	1.02	1.59	0.57	0.38
	A	0.4848	0.4552	0.05832	0.45	0.58	0.14	0.09
	D	0.5354	0.5176	0.08858	0.42	0.66	0.24	0.15

**Table 2:** Kruskal–Wallis test results.

Test Statistics ^a,b^			
	Chi-Square	df	Asymp. Sig.
Goblet5	7.715	2	0.021
Goblet14	9.013	2	0.011
Goblet28	9.073	2	0.011
ETI5	8.180	2	0.017
ETI14	6.020	2	0.049
ETI28	12.500	2	0.002
STI5	9.500	2	0.009
STI14	7.280	2	0.026
STI28	9.780	2	0.008

^a^ Kruskal–Wallis test. ^b^ Grouping variable: Tip_cod.
